# Long-Term Outcome of Repetitive Transcranial Magnetic Stimulation in a Large Cohort of Patients With Cocaine-Use Disorder: An Observational Study

**DOI:** 10.3389/fpsyt.2020.00158

**Published:** 2020-02-28

**Authors:** Graziella Madeo, Alberto Terraneo, Stefano Cardullo, Luis J. Gómez Pérez, Nicola Cellini, Michela Sarlo, Antonello Bonci, Luigi Gallimberti

**Affiliations:** ^1^Novella Fronda Foundation, Padua, Italy; ^2^Department of General Psychology, University of Padua, Padua, Italy; ^3^Padova Neuroscience Center, University of Padua, Padua, Italy; ^4^Global Institutes on Addictions, Miami, FL, United States

**Keywords:** cocaine use disorder (CocUD), transcranial magnetic stimulation (TMS), left dorsolateral prefrontal cortex (l-DLPFC), long-term follow up, addiction

## Abstract

**Background:** Cocaine is a psychostimulant drug used as performance enhancer throughout history. The prolonged use of cocaine is associated with addiction and a broad range of cognitive deficits. Currently, there are no medications proven to be effective for cocaine-use disorder (CocUD). Previous preliminary clinical work suggests some benefit from repetitive transcranial magnetic stimulation (rTMS) stimulating the prefrontal cortex (PFC), involved in inhibitory cognitive control, decision-making and attention. All published studies to date have been limited by small sample sizes and short follow-up times.

**Methods:** This is a retrospective observational study of 284 outpatients (of whom 268 were men) meeting DSM-5 criteria for CocUD. At treatment entry, most were using cocaine every day or several times per week. All patients underwent 3 months of rTMS and were followed for up to 2 years, 8 months. Self-report, reports by family or significant others and regular urine screens were used to assess drug use.

**Results:** Median time to the first lapse (resumption of cocaine use) since the beginning of treatment was 91 days. For most patients, TMS was re-administered weekly, then monthly, throughout follow-up. The decrease in frequency of rTMS sessions was not accompanied by an increase in lapses to cocaine use. Mean frequency of cocaine use was <1·0 day/month (median 0), while serious rTMS-related adverse events were infrequent, consistent with published reports from smaller studies.

**Conclusions:** This is the first follow-up study to show that rTMS treatment is accompanied by long-lasting reductions in cocaine use in a large cohort.

## Introduction

Cocaine is a psychostimulant drug generally used as enhancer of cognitive performances, confidence, sociability, energy, and wakefulness. However, cocaine has been a focus of attention on a global scale for the serious harms related to its use, including addiction and cognitive dysfunctions. Currently, no medications have been proven to be effective for cocaine use disorder (CocUD). The traditional strategy has been to develop medications or psychological interventions to attenuate drug reward, which is mainly mediated by the dopaminergic pathway from the ventral tegmental area (VTA) to the nucleus accumbens. This approach has not resulted in effective therapeutic interventions for cocaine addiction. Recently, repetitive transcranial magnetic stimulation (rTMS) has received increasing attention as a potential treatment for CocUD (1). As we and others have suggested, rTMS of left dorsolateral prefrontal cortex (l-DLPFC) may represent a human translation of preclinical findings that cocaine-seeking is attenuated by optogenetic activation of specific prefrontal circuits (2, 3). Both preclinical and clinical findings suggest that DLPFC has a key role in top-down modulation of emotional and behavioral processes relevant to addiction (4, 5). Thus, exogenously increasing the neuronal excitability of DLPFC via high-frequency rTMS might help reduce craving and prevent lapse^*^^1^ to cocaine use.

Clinical confirmation that rTMS is effective for CocUD awaits results from randomized controlled trials that are now in progress. Studies published to date are limited by small sample sizes and short duration of follow-up. In almost half the published studies using rTMS or a similar intervention for CocUD, there was, strictly speaking, no follow-up: responses (e.g., cocaine craving) were assessed only within the laboratory on the day of stimulation (6–9). In 6 studies that assessed real-world outcomes, follow-up durations ranged from 5 days to 6 months (median 39 days), and the number of cocaine users receiving stimulation ranged from 6 to 36 (median 14) (10–15).

Here we report results from a cohort of 284 cocaine users who received rTMS and were then followed for up to 2 years, 8 months (median 164 days). This is a retrospective chart review, with all the attendant limitations thereof. However, it is also unprecedented in its size and duration. Until there are results from large randomized trials, the data we report here provide the strongest evidence to date that rTMS is well-tolerated and possibly effective in people with CocUD.

## Methods

### Study Design and Participants

Patients signed informed consent on the day of clinic intake and agreed that their data could be used for research. Patients were informed that the data collected would be processed in accordance with the law on privacy and in compliance with Legislative Decree No. 196 of June 30, 2003, “Personal Data Protection Code,” ensuring anonymity. The data were extracted from patient clinical records and anonymized for analysis. All subjects gave their informed consent for inclusion before they participated in the study. This was a retrospective chart review of data from 284 men and women who were treated from 2013 to 2017 and followed for at least 12 weeks after the first week of rTMS sessions. The protocol, limited to the retrospective chart review, was approved by the Ethical Committee for the Psychological Research, Departments of Psychology, University of Padua (Protocol 2551) and the study was conducted in accordance with the Declaration of Helsinki. The total of 284 includes 58 patients who were lost to follow-up within the first 12 weeks (i.e., this was an intent-to-treat sample) but excludes 44 patients for whom 12 weeks had not yet elapsed when data analysis started. All patients voluntarily underwent treatment for CocUD in an rTMS protocol at a clinic center for addiction treatment in Padua, Italy. At intake, each patient was assessed by a psychiatrist and a psychologist with expertise in the treatment of addiction. A complete family, physiological, remote pathological and near pathological history was collected, in addition to a detailed psychiatric, toxicological and clinical history. Patients were between 18 and 70 years of age and met DSM-5 criteria for CocUD. A published screening form was administered to all the patients to exclude contraindications to rTMS (16). Each patient underwent rTMS using a medical device (MagPro R30) targeting the l-DLPFC. The stimulation parameters, in accord with international recommendations for patient safety and ethics (16), were: frequency 15 Hz, intensity 100% of the motor threshold, 60 impulses per stimulation train, inter-train interval 15 s, and 40 total trains, for a session duration of 13 min. For the first 5 days, patients received two rTMS sessions per day (on either an inpatient or outpatient basis, reflecting the patient's needs). rTMS was then administered on an outpatient basis at weekly intervals (twice per day on each session day) for 11 consecutive weeks, as in our published pilot study (15). rTMS was re-administered throughout follow-up on an individualized basis to patients who reported lapses to cocaine use, and to patients whose clinical evaluations showed ongoing cocaine craving, including stress-induced craving.

### Measures

The primary outcome measure was lapse to cocaine use during follow-up. Cocaine use was assessed through a combination of urine screening, self-report, and reports by collateral informants (typically family members). As in our published pilot study (15), the “zero” day for follow-up monitoring was set at 8 days after the initial 5-day course of rTMS (for consistency in outcome analysis, this was done regardless of whether the initial sessions were inpatient or outpatient.) After that 8-day grace period (during which only 29 of 284 patients tested positive for cocaine), any indications of cocaine use (whether by urine or by report) was coded as a lapse. Of the 284 patients, 147 maintained regular contact with the clinic through follow-up visits and phone calls, allowing us to reliably trace their precise patterns of cocaine use and abstinence during follow-up. For the other 137 patients, we have reliable data only on the date of initial lapse to cocaine use (if any) or loss to follow-up.

### Statistical Analysis

Because this is a retrospective chart review, results are presented descriptively. For the sample as a whole, we used Kaplan-Meier survival analysis (SAS Proc Lifetest) to calculate the median number of days until the first lapse to cocaine use. Data were coded as right-censored for patients who were still abstinent at the end of monitoring (~44% of censored cases) or with whom the clinic lost contact (~56% of censored cases). To help contextualize the “first lapse” findings, we display them together with historical control data from an outpatient cohort of 173 cocaine users in the US who were undergoing group and/or individual psychotherapy after discharge from inpatient treatment (17, 18)^2^. The two samples are not intended to be directly comparable, but they share important characteristics: both had just been discharged from an inpatient stay, and both continued to receive treatment as needed during a lengthy outpatient follow-up. For the subset of our 147 patients who were regularly followed, we created a case-by-case display of the relationship between booster sessions of rTMS and stretches of abstinence from cocaine. We know of no published data set to which this can be compared.

### Role of the Funding Source

The funders of the study had no role in study design, data collection, data analysis, data interpretation, or writing of the report. The corresponding author had full access to all the anonymized data in the study and had final responsibility for the decision to submit for publication.

## Results

### Patient Characteristics

Demographic and drug-use data are shown in Table 1. Most patients were male and were using cocaine at least weekly (typically by nasal insufflation, though in some cases by smoking) before initiation of rTMS.

**Table 1:** Sample characteristics.

	**Total sample (*n* = 284)**	**Closely followed subsample (*n* = 147)**
**Age (mean, SD)**	38.3 (8.4)	36.6 (7.7)
**Sex**		
Male	268 (94%)	139 (95%)
Female	16 (6%)	8 (5%)
Cocaine use before treatment entry^*^		
Daily	45%	30%
Weekly or more (not daily)	45%	51%
Monthly or more (not weekly)	2%	5%
Less than monthly	7%	14%
Cocaine route of administration^*^		
Snorting	90%	86%
Smoking	9%	11%
Both	1%	3%

* *Cocaine-use data were available for 126 participants, 43 of whom were in the closely followed subsample; these are the denominators for the percentages. Some percentages add up to slightly <100 due to rounding error*.

### First Lapse to Cocaine Use in the Sample as a Whole

**Figure 1:**
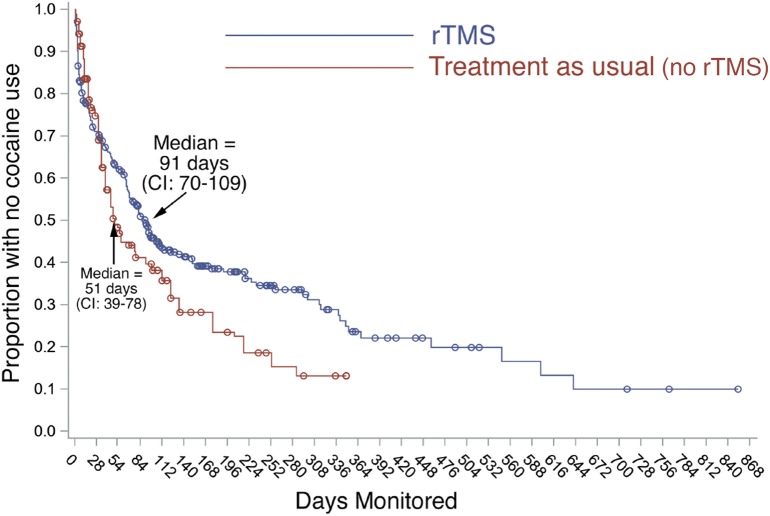
Time to first resumption of cocaine use in full sample and comparison cohort. Blue line: Proportion of our patients (*n* = 284) remaining abstinent from cocaine after the first course of rTMS, monitored by urine screening, self-report, and family corroboration. Day 0 is 8 days after the initial course of rTMS. rTMS continued during follow-up (not shown in this figure). Red line: Proportion of patients in a separate cohort of 173 “treatment as usual (no-rTMS)” outpatients in New Haven, CT (17). Like our rTMS patients, they achieved initial abstinence and were followed up during ongoing treatment (group and individual psychotherapy) for cocaine-use disorder.

For the 284 patients in the whole sample, the duration of follow-up ranged from 4 to 989 days (2 years, 8 months); median was 164 days (just over 5 months). Time to the first lapse is shown in Figure 1. Median time to the first use of cocaine was 91 days (95% confidence interval 70–109 days). Of the patients who had at least 12 months of follow-up, 10 out of 55 (18%) maintained abstinence throughout. Of the patients who had at least 18 months of follow-up, 2 out of 6 (33%) maintained abstinence throughout. In a separate cohort of “treatment as usual” outpatients, median time to the first use of cocaine was 51 days (95% confidence interval 39-78 days). The difference between “treatment as usual” patients and rTMS patients seemed to emerge most clearly around 80 days after discharge from inpatient treatment.

### Patterns of Cocaine Use and Abstinence in the Closely Followed Subsample

**Figure 2:**
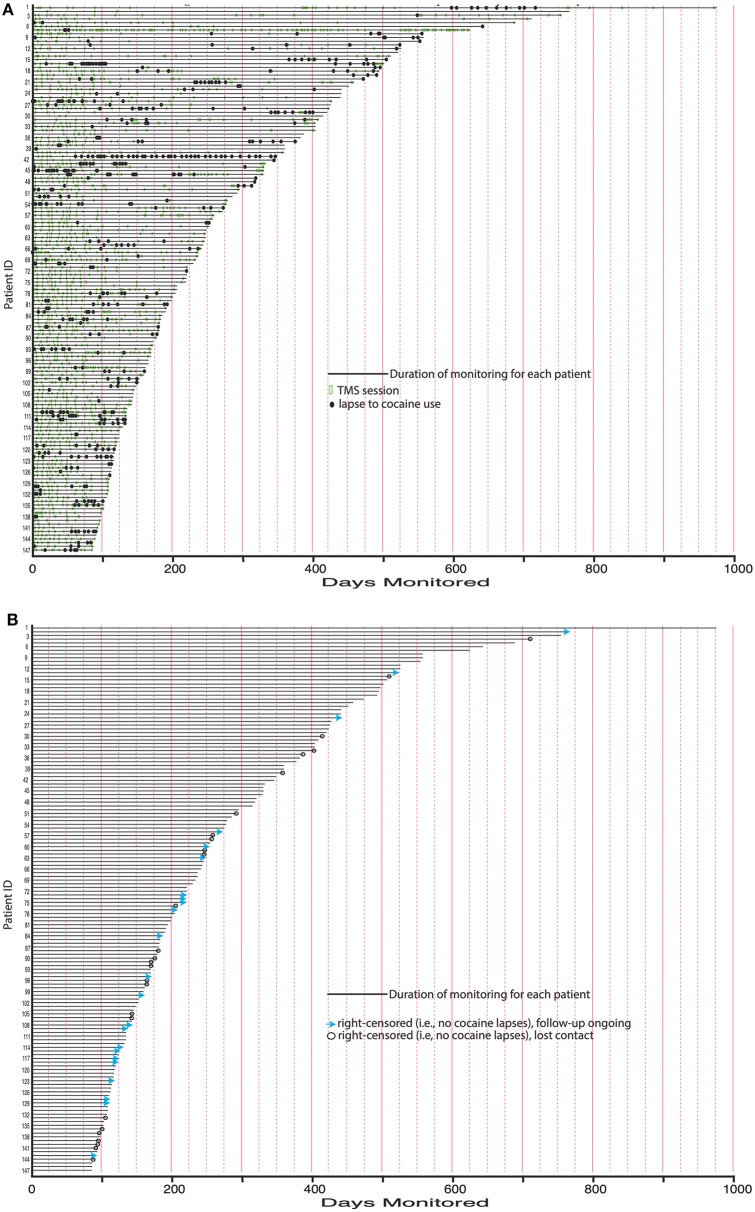
**(A)** Maintenance rTMS sessions and time between lapses for closely followed subsample. Green rectangles: Maintenance rTMS sessions after the initial 8-day course of rTMS. For most patients, rTMS was readministered weekly, then monthly. This was done in response to lapses and in anticipation of lapses. Black circles: Lapses to cocaine use. Lapses tended to occur approximately every month for most patients, but with long stretches of abstinence separating them. **(B)** Causes of censoring in closely followed subsample. Blue arrows: Continuously abstinent patients for whom follow-up is ongoing. Open black circles: Continuously abstinent patients who were lost to further follow-up.

**Figure 3:**
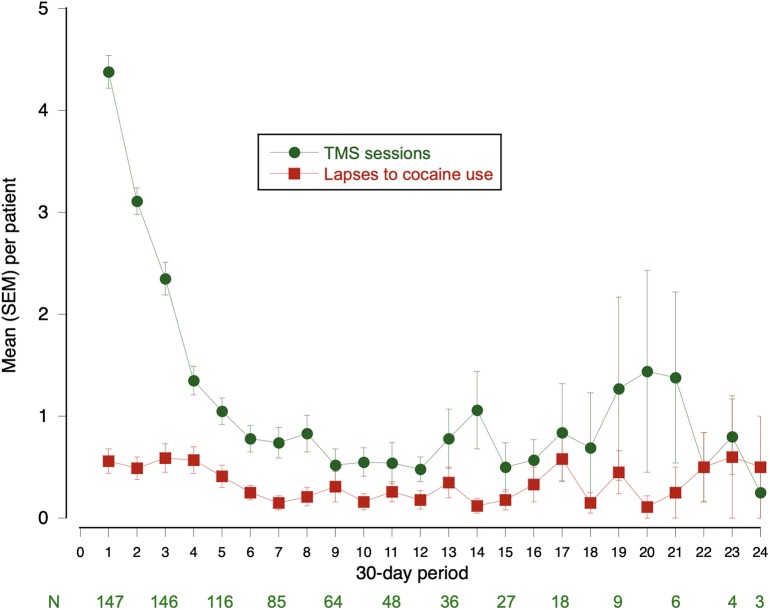
Maintenance rTMS sessions and lapses in closely followed subsample, month by month. This is a collapsed view of data from Figure 2. Green line: Mean (SEM) number of rTMS sessions per patient. Red line: Mean (SEM) number of cocaine lapses per patient.

In the 147 closely followed patients, the duration of follow-up ranged from 84 days (12 weeks) to 974 days (2 years, 8 months); median was 217 days (just over 7 months). Time courses of rTMS and cocaine use are shown in Figure 2. For most patients, rTMS (light rectangles) was re-administered weekly, then monthly. Lapses to cocaine use (black circles) tended to occur every month or so for most patients, but there were long stretches of abstinence between lapses. This is shown in collapsed form in Figure 3, which illustrates more clearly that the gradual decrease in re-administration of rTMS (green circles) did not leave patients more vulnerable to lapses to cocaine use (red rectangles). The mean number of cocaine uses per patient was <1·0 day/month (median 0). Self-reported use of other drugs, including alcohol, gave no indication that patients were substituting other drugs for cocaine (data not shown).

### Adverse Events

Adverse events (AEs) were reported by 41 of the 284 patients. No patient reported more than one. AEs reported were: headache (*n* = 23), hypomania (*n* = 4), anxiety (*n* = 2) irritability (*n* = 2), dental pain (*n* = 2), scalp discomfort during the first 2 weeks of sessions (*n* = 1), angioedema and urticaria (*n* = 1), distractibility (*n* = 1), dizziness (*n* = 1), nausea (*n* = 1), nausea and numbness (*n* = 1), seizure (*n* = 1), and a hypomanic episode (*n* = 1). The seizure occurred in a 27-year-old woman 66 days after her first rTMS session. She has used cocaine shortly before; she had not recently undergone rTMS. The hypomanic episode occurred in a 37-year-old man, just under 90 days after his first rTMS session. His family reported that he had begun speaking aloud to himself without realizing it. rTMS was suspended for medical examinations, which did not show any abnormalities. Other AEs were transient and resolved spontaneously or with over-the-counter medications.

## Discussion

This large set of outcome data adds to the evidence that rTMS can be used to treat CocUD. Our data have all the limitations inherent in retrospective chart review, but one conclusion we can draw confidently is that rTMS for CocUD, at least as administered here, can be considered a long-term rather than only an acute treatment. Several published discussions have stated or implied that rTMS might be a time-limited treatment with lifelong “normalizing” effects on addiction to cocaine or other drugs (19–21). Our findings do not rule that out; we tested only one brain site (left DLPFC) and only one set of stimulation parameters. But at that site, using those parameters, it was feasible, acceptable, and often seemingly necessary to continue treatment sessions p.r.n. for months or years, with adverse events generally few and transient. A similar long-term role has been proposed for rTMS in treatment of mood disorders (22–24). Therefore, rTMS for CocUD may find its place as an additional tool in settings where psychotherapeutic or behavioral treatments are administered. Meta-analyses have repeatedly shown that the most effective known treatments for CocUD are behavioral ones incorporating both tangible incentives for abstinence and social reinforcement of abstinence (25). rTMS could readily be integrated into those behavioral approaches, to be given as needed. Although our data do not permit strong conclusions about the effectiveness of rTMS, it is intriguing to see our outcomes side by side with outcomes in the most comparable cohort we could find. Both cohorts were receiving ongoing care as needed after becoming abstinent from cocaine. The survival curves for resumption of cocaine use indicate a considerably longer duration of abstinence in our rTMS-treated cohort than in the cohort that received “treatment as usual (no-rTMS)” in the form of individual and group psychotherapy.

To our knowledge, there are no available studies in the literature analyzing the lapse rates related to other conventional forms of treatment (pharmacological, pharmaco-, or psychotherapy) for cocaine addiction, especially when considering large cohorts of patients and long period of observation. Nevertheless, we can argue that clinical outcomes, including lapse rate, may show a significant difference compared to conventional treatment for addiction. Compelling evidence from preclinical and clinical studies indicates that rTMS influences neural activity in the short and long term by mechanisms involving neuroplasticity and resulting in substantial behavioral changes (2, 3, 26, 27). These rTMS mediated effects have offered a neural circuit-based treatment for cocaine addiction. Indeed, the long-term neurophysiological changes induced by rTMS on frontal brain regions have the potential to affect behaviors related to drug craving, intake, and relapse and have been proposed as a significant biomarker for predicting treatment outcome.

Human laboratory studies with rTMS suggest that the site we stimulated, left DLPFC, might also be an appropriate target for people with addictions to heroin (28), methamphetamine (29, 30), nicotine (31), or cannabis (32).

In conclusion, rTMS continues to show promise as the first neurobiological treatment for CocUD. Our data add appreciably to the number of patients tested and the length of follow-up. The crucial next step, already under way (e.g., ClinicalTrials.gov identifiers NCT03607591, NCT03333460, and NCT02986438), is represented by sham-controlled randomized trials with sufficient sample size and follow-up duration (33).

## Data Availability Statement

The datasets for this article are not publicly available to protect proprietary information. Requests to access the datasets should be directed to LGa (luigigallimberti.novellafronda@gmail.com).

## Ethics Statement

This study was conducted in accordance with the Declaration of Helsinki, and the protocol was approved by the Local Ethics Committee of University of Padua (Protocol 2551, number code: A0A52E7461375325ABBC1C2D9C54F844). The patients/participants provided their written informed consent to participate in this study.

## Author Contributions

AT and LGa: conceptualization. GM, SC, and LGó: data curation. SC: formal analysis. SC, GM, and LGó: methodology. AT: project administration. LGa: supervision. GM, AT, SC, LGó, NC, MS, and LGa: validation. GM, SC, LGó, NC, MS, and LGa: visualization. GM: writing—original draft. GM, AT, SC, LGó, NC, MS, AB, and LGa: writing—review and editing.

### Conflict of Interest

The authors declare that the research was conducted in the absence of any commercial or financial relationships that could be construed as a potential conflict of interest.

## Footnotes

^1^The terms *lapse* and *relapse* each refer to a resumption of drug use during some period of deliberate abstinence. A lapse is *any* such resumption of drug use, however brief; a relapse is a resumption that is prolonged and problematic. Neither term has a standardized operational definition. In this paper, we refer to any resumption of cocaine use as a lapse. Thus, some of the lapses in our participants could probably be characterized as relapses.

^2^The results from the comparison cohort were collected ~15 years ago as part of a larger data set. The authors who graciously allowed us to use the lapse results were unable to retrieve raw data such as the demographics of the cocaine-using subgroup. In the full comparison sample (*n* = 827), which included people in treatment for dependence on alcohol, marijuana, and heroin, 24% were women, and race/ethnicity were more heterogeneous than in our patients: 47% were European American, 40% were African American, another 12% were classified as Hispanic, and the race of the remaining 1% was classified as “other.” Their mean age was 30. For more information, see Dodge et al. (17).
